# Exposure to PM 2.5 and Birth Outcomes: Evidence from a Population-wide Database.

**DOI:** 10.23889/ijpds.v7i3.1808

**Published:** 2022-08-25

**Authors:** Neil Rowland, Babak Jahanshahi, Duncan McVicar, Dermot O'Reilly, Mark McGovern

**Affiliations:** 1 Queen's University Belfast; 2 Rutger's University

This paper links localised annual average ambient air pollution data to a population database of births in Northern Ireland between 2011 and 2017 to investigate the relationship between particulate matter (PM) 2.5 exposure during pregnancy and a range of birth outcomes, with a focus on birth weight.

Linear regression analysis is used to estimate the effect of exposure to PM 2.5 during pregnancy on commonly studied markers of infant health (birth weight, preterm birth), less commonly studied markers such as Apgar scores, and markers of placental health which potentially represent a mechanism through which pollution might negatively affect newborns. In addition to maternal characteristics and weather conditions, detailed adjustment is made for area of residence. Moreover, comparisons of birth outcomes are drawn amongst siblings born at different times and subject to different levels of in utero exposure, permitting adjustment for mother-specific factors common to siblings.

Most birth outcomes exhibit a strong unadjusted association with PM 2.5 exposure that conforms with expectations. For birth weight, the negative effect of PM 2.5 weakens slightly after adjusting for differences in maternal characteristics and weather, but weakens much further after adjusting additionally for area of residence. After adjustment, being born to mothers in the middling (6-10 micrograms per cubic metre) and highest (10-16 micrograms per cubic metre) categories of exposure is associated with a 12 gram and 32 gram reduction in mean birth weight, respectively, compared to being born into the lowest (3-6 micrograms per cubic metre) category. However, after adjusting additionally for mother-specific factors, these effects become statistically no different from zero. This holds for other outcomes, including measures of placental health.

We find little evidence that exposure to PM 2.5 is related to worse infant health once we adjust as fully as possible for omitted variable bias. We conclude that the association between PM 2.5 and birth outcomes in this population at least partly reflects unmeasured characteristics of families.

